# FACTORS AFFECTING THE IMPLEMENTATION OF THE PRITAH INTERVENTION: LESSONS LEARNED

**DOI:** 10.1093/geroni/igae098.3413

**Published:** 2024-12-31

**Authors:** Klarissa Ponstein, Michel Bleijlevens, Petra Erkens, Jan Hamers

**Affiliations:** Maastricht University, Maastricht, Limburg, Netherlands; Maastricht University, Maastricht, Limburg, Netherlands; Maastricht University, Maastricht, Limburg, Netherlands; Maastricht University, Maastricht, Limburg, Netherlands

## Abstract

The implementation of effective healthcare interventions into routine practice is challenging and influenced by various factors, including the context of implementation, individuals involved, and intervention characteristics. This study aims to explore challenges by implementing the Prevention and Reduction of Involuntary Treatment At Home (PRITAH) intervention. After a careful preparation phase, we faced serious challenges that temporarily delayed implementation. To gain insights into the reasons and factors that led to implementation delay of PRITAH, two separate focus groups were conducted with 1) two policy staff members, a district nurse, two case managers dementia, and a middle manager, and 2) two senior managers and three directors. We examined both barriers and facilitators using the Consolidated Framework for Implementation Research (CFIR). Results show that relevant stakeholders, including members from focus group one, were not involved in a timely manner. Moreover, communication gaps between senior management and middle management, policy staff, district nurses and dementia case managers led to inconsistencies in knowledge of the implementation steps being taken and hindered the decision-making processes. Nevertheless, the members of both focus groups acknowledged the shared goal of preventing involuntary treatment at home as facilitator, emphasizing the importance of goal alignment. Furthermore, a clear implementation plan from the intervention developers ensured that implementation efforts were guided by organizational expectations. The delays in implementing PRITAH emphasize timely stakeholder engagement, effective communication, joint alignment of goals and a clear implementation plan. Based on the results an adapted implementation plan has been developed.

